# Bibliographical

**Published:** 1858-06

**Authors:** 


### Bibliographical.

We have received from the Publishers, Messrs. Blanchard & Lea, of Philadelphia, "Dunglison's Medical Dictionary," revised and greatly enlarged, which is so well known as a *Standard Authority* in Medical Lexicography, that we feel it to be useless to say more than it can be purchased in this city, from Messrs. J. J. Richards & Co., through whose hands it has reached us.

We have also received from the same publishers, through the same hands, "A Manual of Medical Diagnosis," being an analysis of the Signs and Symptoms of Disease; by A. W. Barclay, M. D., Fellow of the Royal College of Physicians, &c.

We would desire to commend this work to the attention of all those who desire to become acquainted with this all-important part of the Science of Medicine.

There are few physicians, even of great and discriminating experience, who will not acknowledge their deficiency in that knowledge which this book is designed to convey; and to those who are just entering the profession, there are few books which can be of more value than one which will guide them correctly in the systematic investigation of the character of disease—upon the correct understanding of which their whole success must, of course, depend.

It is then to the student especially, that we would commend this work as a valuable aid in establishing in his mind, the correct principles upon which Diagnosis should be based.

We are also indebted to the same source, for "*The Principles and Practice of Obstetrics*," by Henry Miller, M. D., Professor of Obstetric Medicine, in the University of Louisville, which we are not now prepared to review, as we believe it deserves. From some little examination we have been able to give the work, our interest has been sufficiently excited to desire to give it a careful reading, which it has not been in our power to do. We hope to be able to notice it more fully upon some future occasion.

We would desire also, to call the attention of the profession to a new edition of the "Elements of Inorganic Chemistry," including the applications of the Science in the Arts, by Thos. Graham, F. R. S., L. & E., late Professor of Chemistry, in University College, London. Edited by Henry Watts, B. A., F. C. S., and Robert Bridges, M. D.

In this work is presented Mr. Graham's Inorganic Chemistry complete—the first portion, up to page 430, having been edited by Dr. Bridges, in 1852; the remainder being reproduced without alteration, from the English Edition, issued a few months since, under the supervision of the author, by Mr. Watts, whose elaborate Supplement will be found to bring the subjects embraced in the first portion, on a level with the most advanced condition of the science. Blanchard & Lea, Phila.

We have also to acknowledge the reception of "Principles of Physiology," designed for the use of schools, academies, colleges, and the general reader, comprising a familiar explanation of the structure and functions of the Organs of Man; illustrated by comparative references to those of the inferior animals; also, an Essay on the Preservation of Health. As an Elementary Treatise, and as one designed for popular use, from a slight examination we think it valuable, and, indeed, think it probable that it is the best of its class. It has been received from, and may be obtained of, Messrs. Samuel S. & W. Wood, 389 Broadway, New York City.

